# Supplementary material for: Middle-way flexible docking: Pose prediction using mixed-resolution Monte Carlo in estrogen receptor α
Source: PLoS One. 2019 Apr 23;14(4):e0215694. doi: 10.1371/journal.pone.0215694 (PMC6478315; doi:10.1371/journal.pone.0215694)
Supplement: S1 Table — (PDF) [file pone.0215694.s001.pdf]

Middle-way flexible docking: Pose prediction using mixed-resolution Monte Carlo in estrogen receptor α: Supporting Information Table S1

Justin Spiriti, Sundar Raman Subramanian, Rohith Palli,
Maria Wu, and Daniel M. Zuckerman*
Corresponding author: zuckermd@ohsu.edu

April 10, 2019

Ligand ID	PDB code for reference structures
0CZ	3UUA
17M	2B1Z
1GJ	4IVW
1GM	4IU7
1GQ	4IUI
1GR	4IV2
1GS	4IV4
1GT	4IVY
1GU	4IW6
1GV	4IWC
2OH	3UU7
458	2B1V
459	2FAI
4OH	3L03
689	1ZKY
DES	3ERD
DRQ	2G5O
EED	2QGT
EI1	2QAB
ESE	4PPS
ESL	3Q95
EST	1QKU
ETC	1L2I
EZT	2P15
FSV	4PPP
GEN	1X7R,2QA8
HZ3	2QR9
J2Z	3HLV
J3Z	3HM1
KN2	2QA6
KN3	4IW8,3OSA
ODE	2QH6
PIQ	2QXM
STL	4PP6
T3O	2G44
ZTW	1GWQ

Table S1: Agonists used in this work.
