# Supplementary material for: Middle-way flexible docking: Pose prediction using mixed-resolution Monte Carlo in estrogen receptor α
Source: PLoS One. 2019 Apr 23;14(4):e0215694. doi: 10.1371/journal.pone.0215694 (PMC6478315; doi:10.1371/journal.pone.0215694)
Supplement: S2 Table — (PDF) [file pone.0215694.s002.pdf]

Middle-way flexible docking: Pose prediction using mixed-resolution Monte Carlo in estrogen receptor α: Supporting Information Table S2

Justin Spiriti, Sundar Raman Subramanian, Rohith Palli,
Maria Wu, and Daniel M. Zuckerman*
Corresponding author: zuckermd@ohsu.edu

April 10, 2019

Ligand ID	PDB code for reference structures
0CZ	3UUA
17M	2B1Z
1GJ	4IVW
1GM	4IU7
1GQ	4IUI
1GR	4IV2
1GS	4IV4
1GT	4IVY
1GU	4IW6
1GV	4IWC
2OH	3UU7
458	2B1V
459	2FAI
4OH	3L03
689	1ZKY
DES	3ERD
DRQ	2G5O
EED	2QGT
EI1	2QAB
ESE	4PPS
ESL	3Q95
EST	1QKU
ETC	1L2I
EZT	2P15
FSV	4PPP
GEN	1X7R,2QA8
HZ3	2QR9
J2Z	3HLV
J3Z	3HM1
KN2	2QA6
KN3	4IW8,3OSA
ODE	2QH6
PIQ	2QXM
STL	4PP6
T3O	2G44
ZTW	1GWQ

Table S2: Agonists used in this work.

Ligand ID	PDB code for reference structures
369	3DT3
AEJ	1XQC
AIH	1XP1
AIJ	1XP9
AIT	1XPC
AIU	1XP6
C3D	2OUZ
CM3	1YIN
CM4	1YIM
DC8	2Q70
E4D	1SJ0
GW5	1R5K
I0G	2I0J
IOG	2IOG
IOK	2IOK
JJ3	2QE4
KN0	3OS8
KN1	3OS9*
L4G	2AYR
LLB	2R6W
LLC	2R6Y
OHT	3ERT,2BJ4,2JF9
PTI	1UOM
RAL	2JFA,2QXS
WST	2POG

Table S3: Antagonists used in this work. *There were two reference structures in this PDB file, which were used separately (see text).
