# Supplementary material for: Middle-way flexible docking: Pose prediction using mixed-resolution Monte Carlo in estrogen receptor α
Source: PLoS One. 2019 Apr 23;14(4):e0215694. doi: 10.1371/journal.pone.0215694 (PMC6478315; doi:10.1371/journal.pone.0215694)
Supplement: S1 Fig — (a), (c), and (e) are for translations; (b), (d), and (f) are for rotations. (a)-(b) Cumulative probability density function of generated move sizes. (c)-(d) Average final acceptance probability of NCMC and MC moves as a function of move size. (e)-(f) Combined acceptance probability (the overall proportion of all NCMC or MC moves that were accepted and of the given size) which is the product of the generating probability. (PDF) [file pone.0215694.s003.pdf]

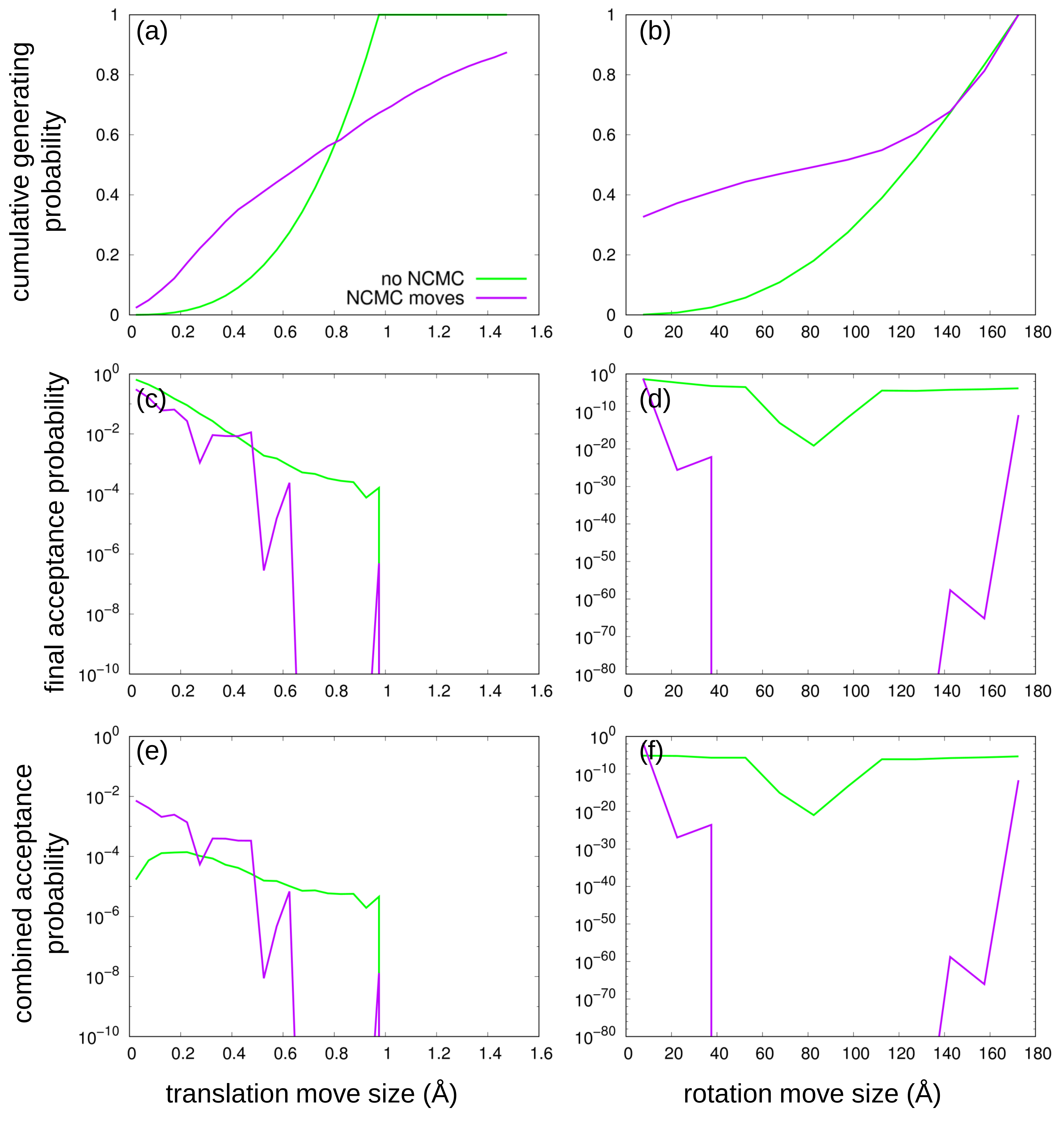

cumulative generating probability
final acceptance probability
combined acceptance probability
(a)
(b)
(c)
(d)
(e)
(f)
no NCMC
NCMC moves
translation move size (Å)
rotation move size (Å)
