# Supplementary figures and images for: Middle-way flexible docking: Pose prediction using mixed-resolution Monte Carlo in estrogen receptor α
Source: PLoS One. 2019 Apr 23;14(4):e0215694. doi: 10.1371/journal.pone.0215694 (PMC6478315; doi:10.1371/journal.pone.0215694)

ligand heavy atom RMSD (Å)

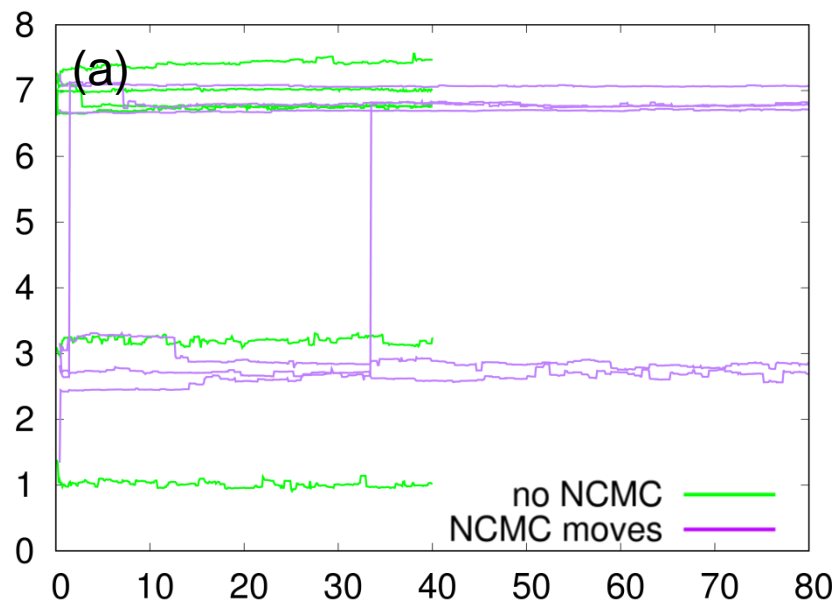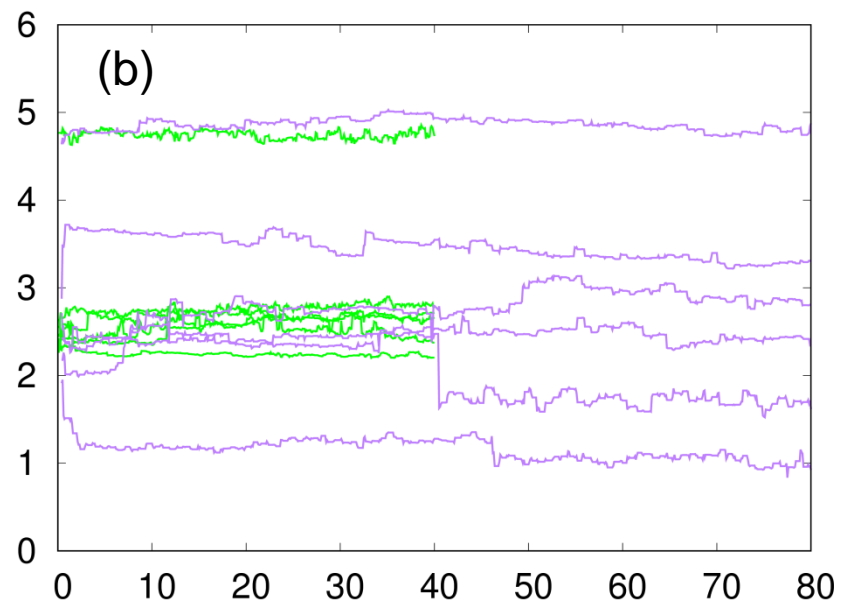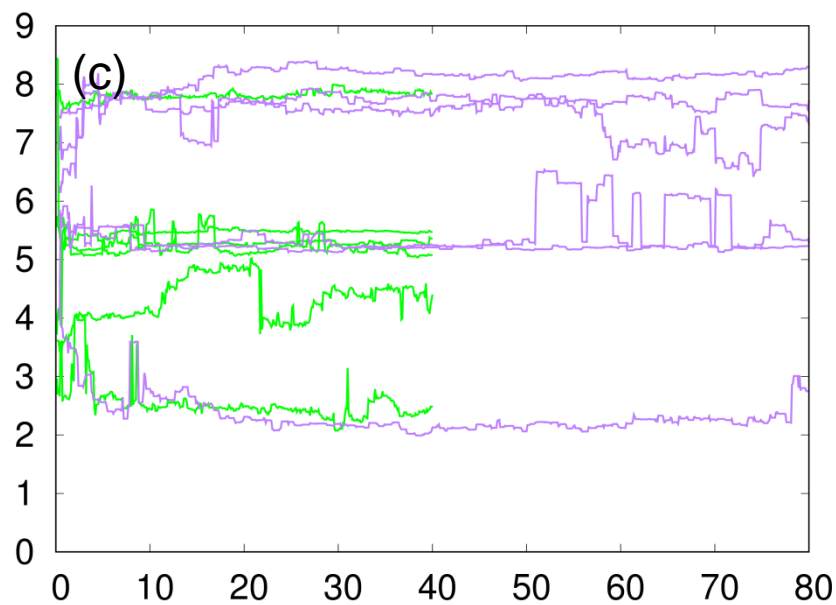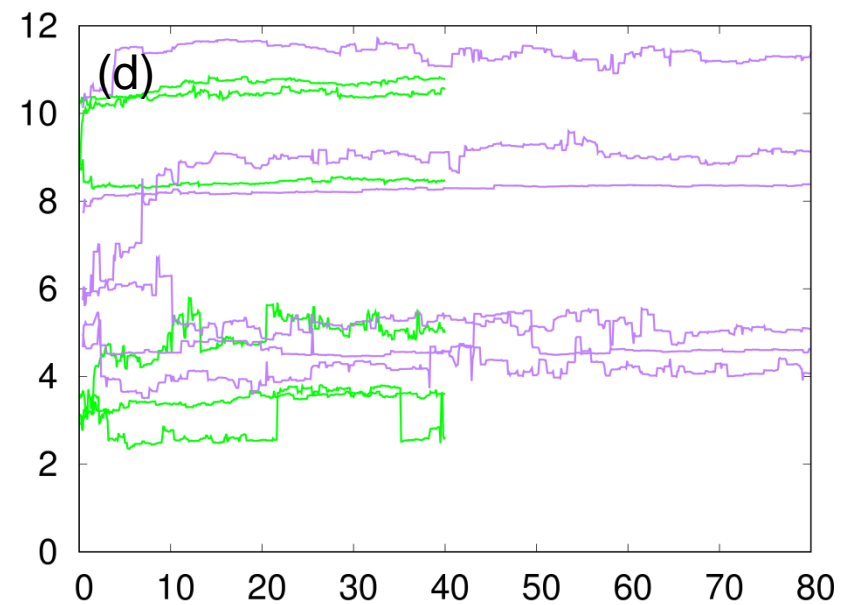

$10^3$  trial moves

Supplement: S2 Fig — (a) genistein; (b) diethylstilbestrol; (c) raloxifene; (d) the drug AIU. Six out of 120 docking runs are shown for each drug. (PDF) [file pone.0215694.s004.pdf]
